# Extensive sequence turnover of the signal peptides of members of the GDF/BMP family: exploring their evolutionary landscape

**DOI:** 10.1186/1745-6150-4-22

**Published:** 2009-07-16

**Authors:** Reiner A Veitia, Sandrine Caburet

**Affiliations:** 1 Institut Jacques Monod, UMR 8104, CNRS, Paris, France; 2 Université Paris-Diderot/Paris 7, Paris, France

## Abstract

We show that the predicted signal peptide (SP) sequences of the secreted factors GDF9, BMP15 and AMH are well conserved in mammals but dramatic divergence is noticed for more distant orthologs. Interestingly, bioinformatic predictions show that the divergent protein segments do encode SPs. Thus, such SPs have undergone extensive sequence turnover with full preservation of functionality. This can be explained by a pervasive accumulation of neutral and compensatory mutations. An exploration of the potential evolutionary landscape of some SPs is presented. Some of these signal sequences highlight an apparent paradox: they are encoded, by definition, by orthologous DNA segments but they are, given their striking divergence, examples of what can be called functional convergence.

Reviewers:

This article was reviewed by Fyodor Kondrashov and Eugene V. Koonin.

## Findings

A typical signal peptide (SP) involves a hydrophobic alpha-helical region which is called the h-region. This hydrophobic segment is generally shorter (i.e. approximately 7–15 residues) than required for a transmembrane helix. The h-region is close the N-terminus of the protein but it is generally preceded by a slightly positively charged n-region which is variable in length (i.e. 1–12 residues). The cleavage site for the signal peptidase lies between the h-region and a c-region, a stretch of 3–8 amino acid involving rather polar and uncharged amino acids [1,2].

TGFβ superfamily members are secreted proteins that play important roles in developmental and physiological processes in mammals and other organisms. They are classified into the TFGβ/Nodal/Activin group and the BMP/GDF group [3-7]. In our analysis, we will focus on the SPs of some members of the BMP/GDF group, namely BMP15, GDF9 and AMH. BMP/GDF factors are synthesized as inactive precursors (pre-proproteins). They comprise an N-terminal SP, a propeptide and a mature region located at the C-terminal part of the protein. Thus, production of the mature bioactive polypeptide requires extensive post-translational processing: SP removal, dimerisation and further cleavage [8,9].

**Figure 1:**
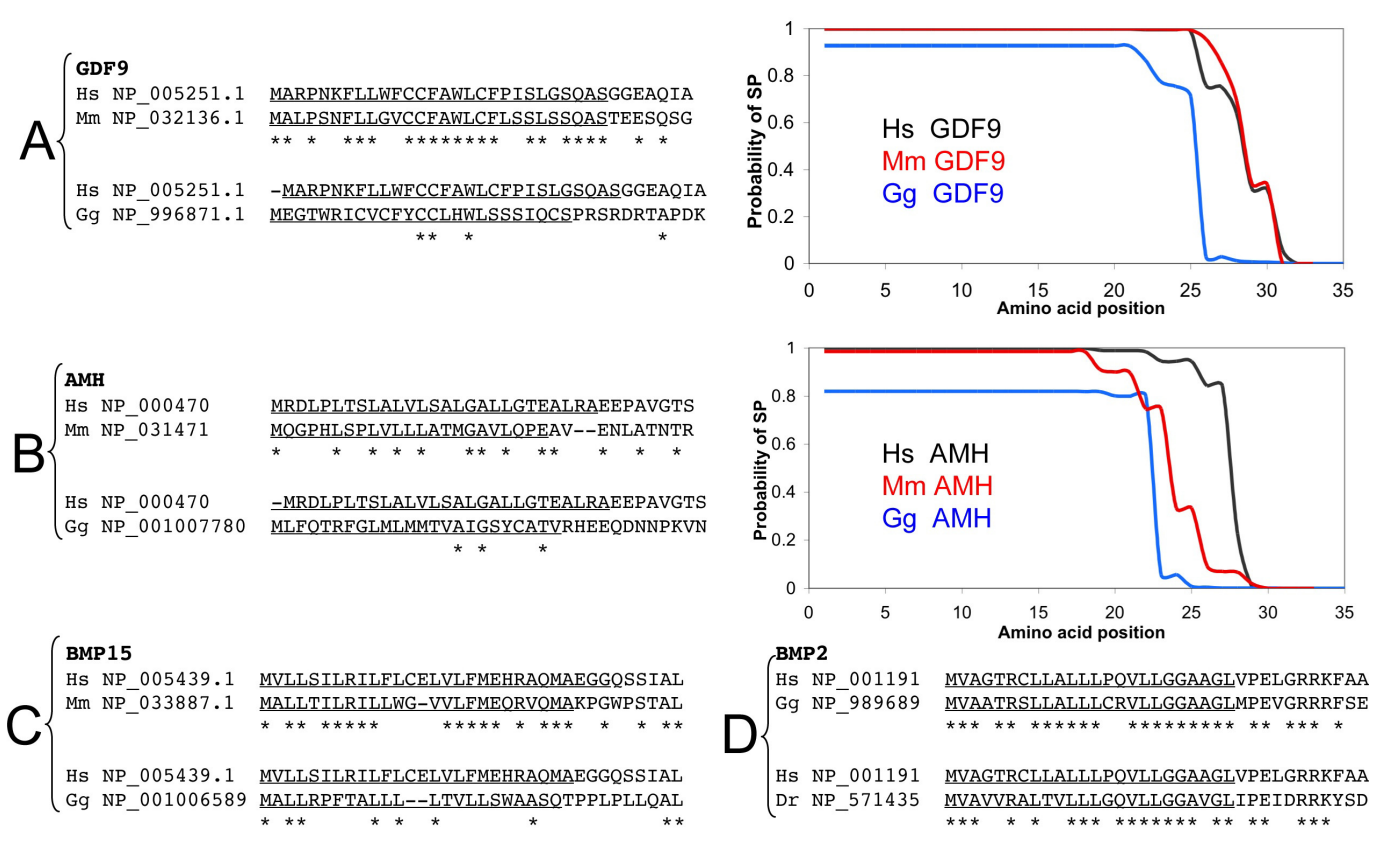
**Signal peptides in some members of the BMP/GDF family are highly divergent**. A. Alignment of human (*Homo sapiens*, Hs), murine (*Mus musculus*, Mm) and chicken (*Gallus gallus*, Gg) N-terminal GDF9 sequences. *Bona fide *predicted signal peptides are underlined. Asterisks represent conserved amino acid positions. The right panel shows the predicted probability of being a signal peptide for each sequence, according to Phobius (using the sequences provided in the alignments). A clear sequence divergence but functional preservation is obvious for the mammalian and chicken GDF9 sequences. **B**. Alignment and predicted signal peptide score for human, murine and chicken N-terminal AMH sequences. Here sequence divergence is obvious even between man and mouse. **C**. Alignment of the human, murine and chicken N-terminal BMP15 sequences. Again sequence divergence is observed between mammals and chicken, with functional conservation, as noticed for GDF9. **D**. Alignment of human, murine and chicken N-terminal sequence of BMP2, displaying a high sequence similarity between mammals and chicken and even fishes (*Danio rerio*, Dr).

As shown in Figure 1, the predicted SP sequences of GDF9 and BMP15, and to a lesser degree of AMH, are well conserved in mammals. However, strong divergence is noticed between the mammalian and the chicken orthologs. Nevertheless, bioinformatic predictions of SP using Phobius [10] and SignalP [11] clearly show that the divergent protein segments do encode SPs (Figure 1A, 1B and 1C). Divergence is even stronger in comparisons with sequences from cold-blooded vertebrates (i.e. fishes, data not shown). However, this behavior is not a strict rule within this family, since, for instance, the SP of BMP2 is very well conserved between mammals and chicken (Figure 1D and Table 1), and even between mammals and fishes (i.e. *Danio rerio*/zebrafish). Strong sequence divergence in the SP of AMH, BMP15 and GDF9 shows that such sequences can undergo extensive turnover with full preservation of their functionality. This can be explained by the accumulation of multiple neutral and/or intragenic compensatory mutations.

**Table 1:** Percentage of protein sequence identity for proteins of the BMP/GDF family, between human and mouse and between human and chicken, for the total protein and the relevant signal peptide.

	H. sapiens versus M. musculus % sequence identity		H. sapiens versus G. gallus % sequence identity	
	Whole length	Signal peptide	Whole length	Signal peptide
AMH	74.0	40.0	45.1	43.3
BMP2	92.4	93.3	81.3	66.7
BMP3	81.1	63.3	67.2	36.7
BMP4	97.5	100.0	85.3	90.0
BMP5	93.2	86.7	92.7	70.0
BMP6	91.9	96.7	85.1	16.7
BMP7	97.7	100.0	91.4	30.0
BMP9 (GDF2)	80.4	53.3	61.8	30.0
BMP10	85.5	73.3	74.7	46.7
BMP14(GDF5)	92.3	90.0	70.6	33.3
BMP15	64.2	60.0	47.0	13.3
GDF3	71.1	33.3	48.9	33.3
MSTN (GDF8)	96.3	80.0	92.0	53.3
GDF9	74.1	66.7	589	20.0

The percentage of sequence identity for the signal peptide was calculated for the first 30 amino acid in human sequences, excluding gaps. Notice that there are several possibilities, ranging from strong conservation in the three species including the signal peptide (BMP4), passing through cases of overall conservation excluding the signal peptide (GDF8), to cases of rather low conservation in the three species (BMP15).

Wright (1964) and Kimura (1990) have defined compensatory mutations as those masking the deleterious effect of another mutation or as mutations that are independently deleterious but neutral when combined [12,13]. However, this criterion can be relaxed to non deleterious mutations that compensate for the effects of potentially deleterious ones. The most obvious cases of canonical compensatory mutations are provided by alterations affecting the secondary helical structures of tRNA and rRNA molecules, whose effects are counterbalanced by changes restoring base pairing [14,15]. Compensatory mutations in the context of proteins and cis-regulatory sequences are also well known [16].

The analysis of the SP of some members of the TGFβ superfamily is very instructive to understand the evolution of neutral and compensatory mutations in protein coding regions. Although it is a difficult exercise to predict the sequence of events leading to the emergence of several compensated mutations, we will explore this issue with the particularly interesting case of the SP of GDF9 in mammals.

**Figure 2:**
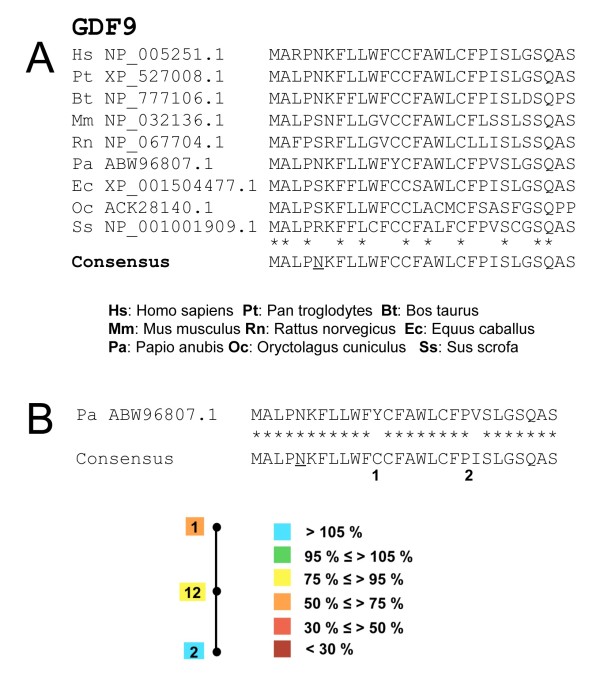
**Consensus sequence and predicted mutation landscape of the signal peptide of GDF9**. **A**. Multiple alignment of 9 mammalian sequences, and definition of the consensus, subsequently used as a reference sequence. The asterisks denote strictly conserved amino acids. Note that in position 5, N and S are equiprobable. **B**. The alignment between the consensus and the *Papio anubis *(Pa) sequence shows that only two positions are divergent. For the sake of simplicity, N is kept at position 5 (underlined), because it is present in the *P. anubis *sequence. The potential impact of the mutations (put in the context of the consensus) on the signal peptide function is represented underneath: a black dot symbolizes each mutation or their combination, and the labels are color-coded according to the signal peptide probabilities predicted by Phobius (as % with respect to the score of the consensus with no mutations, using the sequence length displayed in the figure).

First of all, we need to define a 'reference' sequence, to which compare a sequence in question. For this we have derived a consensus mammalian SP sequence, from a multiple alignment of the N-terminus of GDF9 (Figure 2A). We have assumed that the consensus sequence is likely to be closer to a eutherian ancestral sequence than any sequence considered in isolation. The alignment between the consensus and any individual sequence shows that divergence varies greatly, in nature, position and number (from no divergence for the *P. troglodytes *SP to 8 differences for *O. cuniculus*). To assess whether the divergent sites noticed in the various sequences are either functionally irrelevant (neutral) or compensated, we have explored the predicted 'fitness' landscape by taking into account the functional impact of each change in the context of the consensus sequence, either isolated or in various combinations. Specifically, we used the bioinformatic tool Phobius to predict the SP character of all possible combinations of 'mutations' in the context of the consensus sequence, and by calculating the ratio of the resulting predicted activity over the predicted activity for the consensus sequence. We verified most of the results with SignalP (data not shown).

Figure 2B shows the simplest example of compensatory mutation: in *Papio anubis*, the SP of GDF9 displays only 2 divergences from the consensus sequence. The first one involves a C in the consensus and a Y in the sequence from *Papio anubis*. The 'mutation' C12Y (in the context of the consensus), is predicted to be deleterious when present alone, as it drives down the SP activity to 51% of that of the consensus. The second mutation I21V has a slightly positive effect, yielding 113% of the activity of the consensus sequence (when alone), and is able to compensate the negative impact of the first one, as the two mutations together provide 94% of the consensus activity.

**Figure 3:**
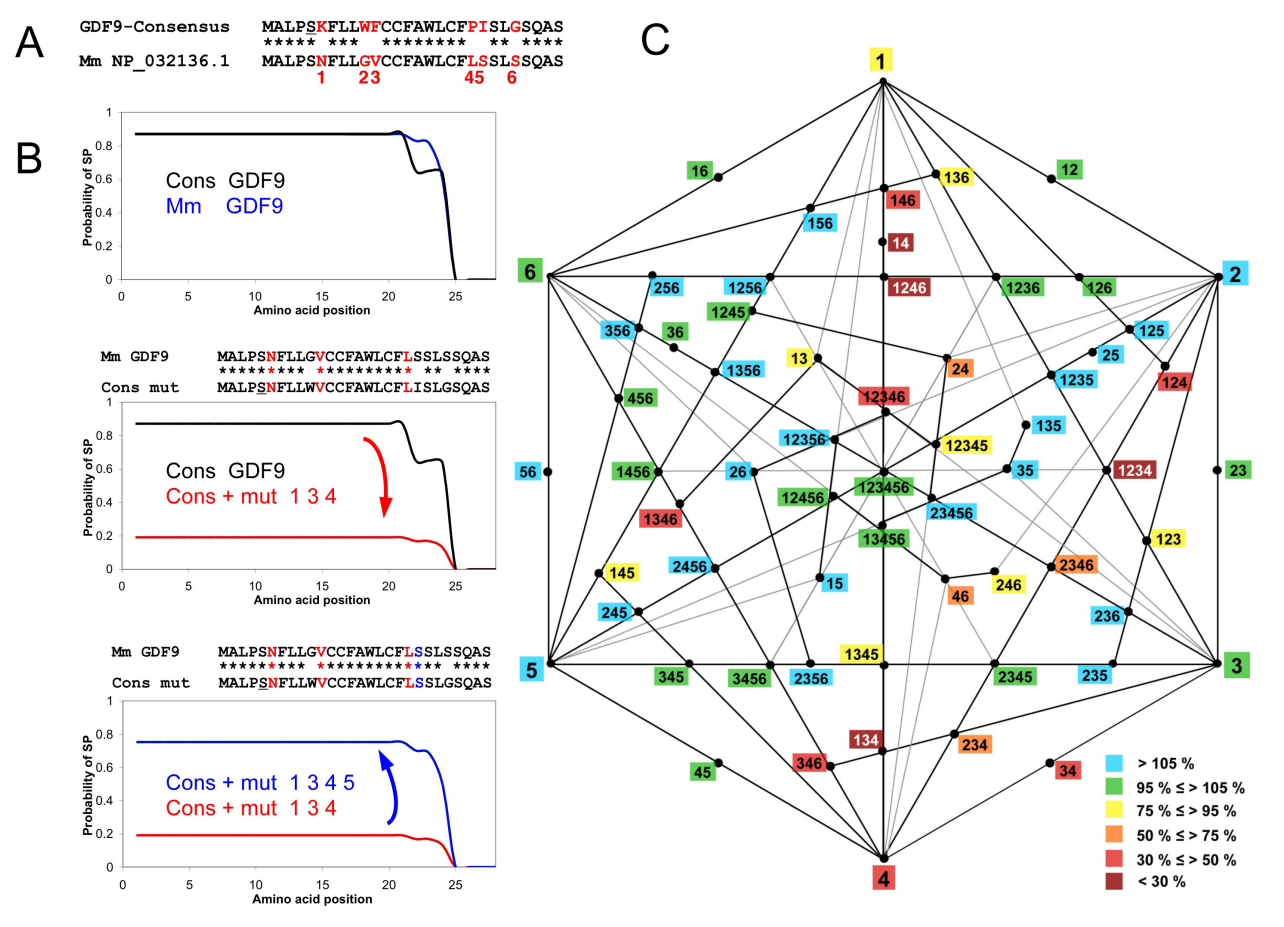
**Analysis of compensatory mutations in the signal peptide of murine GDF9**. **A**. Alignment of consensus and murine (Mm) signal peptide sequences of GDF9. The consensus sequence is the same as presented in Figure 2. The divergences between the two sequences are identified by numbers that are subsequently used in the analysis. **B**. Signal peptide probability of the different sequences, as predicted by Phobius. Top panel: predicted signal peptide probability for the consensus and murine sequences. Middle panel: drastic diminution of the predicted signal peptide probability when introducing 3 of the 6 murine divergent sites in the consensus sequence. Bottom panel: restoration of the signal peptide probability when introducing the I21S (compensatory) mutation. **C**. Diagram depicting all possible combinations of the 6 divergent sites (and their effects when introduced in the consensus sequence). The 6 divergent sites are represented by their numbers, as shown in A, at the vertices of the hexagon. The lines link the mutations together and each black dot at the intersections represents a specific combination. The combination at the center of the hexagon contains all the mutations (i.e. the present state of the sequence in the relevant species). The labels are color-coded according to the signal peptide probabilities predicted by Phobius (as % with respect to the score of the consensus with no mutations). For the murine sequence, the predicted impact varies from slighlty 'hypermorphic' (up to 113% of the score of the consensus) to very deleterious (down to 21% of the score of the consensus).

A completely different landscape is predicted for the murine sequence. The alignment of the consensus and the murine SP sequences shows that 6 positions are divergent (Figure 3A). Now we can ask whether the 6 amino acid changes between the consensus and the murine sequences (numbered from 1 to 6 in Figure 3A) are neutral to function or, more interestingly, compensated. According to Phobius predictions, the consensus sequence for GDF9 should contain a strong SP, and this is also the case for the murine sequence, in spite of the divergent sites (Figure 3B). A very telling example of compensatory mutations is provided by the mutations K6N+F11V+P20L (i.e. mutations 1, 3 and 4 placed in the context of the consensus sequence). Such alterations of the consensus have a negative effect on the SP, since the predicted SP probability is only 21% of the one obtained for the consensus (Figure 3B). How can such a negative effect be compensated? As shown in Figure 3B, a further substitution (I21S, mutation number 5) is able to compensate for the negative impact of K6N+F11V+P20L, restoring almost completely the SP character of the sequence. Similar results are obtained when using SignalP. To explore the fitness landscape more systematically, we have introduced the 64 combinations of mutations in the context of the consensus sequence, and evaluated their functional impact (Figure 3C). We represent the fitness landscape as an hexagon, where each vertex represents a mutation, and the lines joining the vertices (and the intersections of the latter) represent different combinations of mutations. A color code similar to that of Figure 2B reflects the predicted functional impact of a mutation or their combinations. This systematic analysis shows that some combinations are particularly deleterious. For instance, combinations involving mutations 1 and 4 together (in the context of the consensus) are highly problematic, but their effects are compensated by mutation 5. Interestingly, some mutations appear rather 'hypermorphic' with respect to the consensus. This is the case of mutations 5 and 6 (alone or associated) which may foster compensation of potentially deleterious changes.

**Figure 4:**
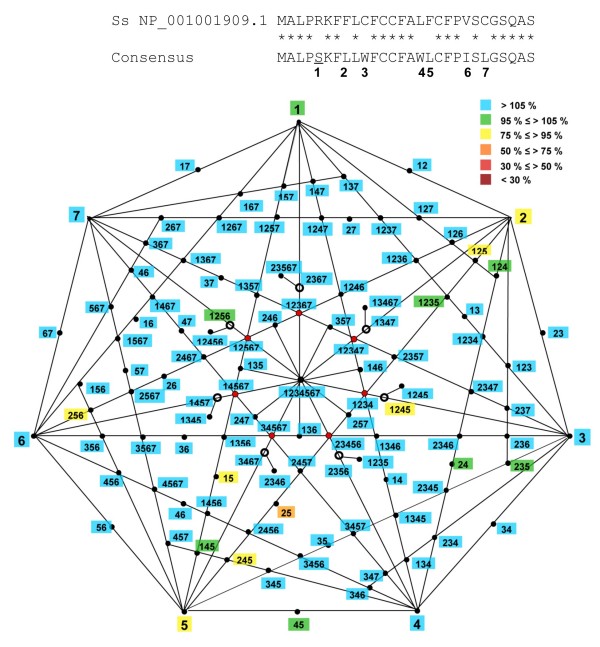
**Predicted neutral mutations in the signal peptide of pig GDF9**. **Upper panel**. The alignment between the consensus and the *Sus scrofa *(Ss) sequence shows that 7 positions are divergent. **Lower panel**. The diagram depicting all possible combinations of these 7 divergent sites (and their effects when introduced in the consensus sequence) is a heptagon. The 7 divergent sites are represented by their numbers, as shown in the alignment. The lines link the mutations together and each black dot at the intersections represents a specific combination. The labels are color-coded according to the signal peptide probabilities (as in previous figures). Green and blue labels, obviously in majority, represent neutral and slightly 'hypermorphic' changes, respectively. For the sake of clarity, a set of 14 combinations were not be placed on the diagram, but they all contained five or six changes each, and all led to at least 109% of the score of the consensus, and would have been color-coded in blue.

A much quieter landscape is predicted for the *Sus scrofa *SP sequence (Figure 4), that presents 7 changes when compared to the consensus (numbered from 1 to 7 in Figure 4). A systematic analysis shows that almost all mutations and their combinations are either neutral or even slightly hypermorphic (in green and blue, Figure 4). Only the second (L8F) and the fifth (L17F) changes are predicted to slightly diminish SP activity. As expected, their combination is predicted to be the most deleterious one, yielding 71% of the consensus activity. Nevertheless, this functional impact is weak, and is very easily compensated by the introduction of any other change. Therefore, this quiet landscape can be considered as representing an evolution of the sequence by fairly neutral mutations.

Given that compensated mutations separately can be disadvantageous and that they are unlikely to appear concertedly, one puzzling aspect is the sequence of steps leading to their appearance. Theoretical studies of compensatory mutations related to RNA secondary structure show that "almost all bases [of the RNA molecule] can be substituted sequentially without ever changing the shape [phenotype] of the molecule" [17]. This is linked to the existence of neutral mutational networks that opens the possibility of changing the genotype while preserving the phenotype [18,19]. In the context of the murine SP of GDF9, the simplest scenario would predict that the first change expected to have appeared in the ancestral murine sequence is I21S (mutation 5) which does not alter SP activity (and might even increase it), and that the other mutations might have appeared later in whatever order, because they are always compensated by S21.

Rapid sequence turnover also bears important practical consequences. For instance, we have recently detected a *BMP15 *mutation leading the potentially damaging variant S5R in a patient with severe ovarian dysfunction [20]. This mutation lies within the SP of BMP15, and very recently, Rossetti et al. found that it decreases significantly the activity/amount of the secreted protein [21]. This is in agreement with our *in silico *analyses using Phobius and SignalPep that predicted a quantitative alteration of SP processing. In order to further assess the potential deleterious effect of this amino acid change we used the SIFT software, which uses protein sequence conservation data and the physicochemical properties of amino acids to calculate the probability for an amino acid substitution of being deleterious [22]. Ser5 in BMP15 is conserved in vertebrates ranging from the zebrafish to mammals. However, the divergent chicken sequence does have an obviously compensated arginine at position 5. Thus, depending on the inclusion or exclusion of the chicken sequence in the alignment, the mutation p.S5R is predicted to be either very pathogenic or not pathogenic at all.

## Speculations

It is known that sex and reproduction-related genes, as is the case of BMP15, GDF9 and AMH [23,24], can have increased evolutionary rates [25]. This might explain at least in part the divergence of the SPs observed here. For some of these SPs, sequence turnover is so important that the original protein segments have been almost entirely replaced by new sequences, fully retaining a SP function. This can be explained by an important accumulation of neutral and compensatory mutations through an evolutionary scale. Thus, sequences encoding SPs deriving from common ancestral sequences (which is obvious from the underlying gene structures and homology outside the SP regions) can be highly divergent at present: they are orthologous by definition but their corresponding encoded peptides are by definition functionally convergent.

## Abbreviations

AMH: Anti-Mulleran Hormon; BMP: Bone Morphogenetic Protein; GDF: Growth-Differentiation Factor; SMAD: Sma- and Mad-related (mothers against decapentaplegic) proteins; SP: Signal Peptide; TGFβ: Transforming Growth Factor, Beta.

## Competing interests

The authors declare that they have no competing interests.

## Authors' contributions

RAV proposed the idea, performed the initial analysis and wrote the paper. SC: performed further analyses, designed the diagrams and wrote the paper. Both authors read and approved the final manuscript.

## Reviewers' comments

### Reviewer's report 1

Fyodor Kondrashov, Centre for Genomic Regulation, Barcelona, Spain

Review of Veitia and Caburet, titled "Extensive turnover of the signal peptides of some members of the GDF/BMP family: whatever happened to these sequences?

This is a beautiful story of the compensatory nature of signal peptide evolution and one of the few attempts out there to actually define the nature of fitness ridges in protein space. Figure 2C is a wonderful depiction of one of the most important questions in macroevolution – how different genotypes are connected in fitness space. The use of a consensus sequence as a rough representative of the ancestral state is one of my favorite ideas and, of course, has its biases, but they should be relatively small if used in a correct phylogenetic setting as has been done here. More works should replicate what Veitia and Caburet have done here.

Specific critiques:

1) I think that the title of the paper does not do justice to the content. I think that the most wonderful result is not the extensive turnover of sequence (we know this must be possible because we see many different highly divergent orthologs) but in the fact that the authors can reproduce the fitness landscape of an entire functional unit.

Authors' response: We agree with the referee and we have changed the title accordingly.

2) The compensation of a deleterious allele by a neutral variant is known as a Dobzhansky-Muller incompatibility, and has been described in a molecular level as compensations of disease mutations.

Authors' response: Dobzhansky-Muller incompatibility has indeed been described in eukaryotic hybrids, and the molecular basis of this incompatibility is thought to be due to mismatches in macromolecular complexes and cellular networks (failure of intergenic compensation). Nevertheless, we failed to link this to our findings, which imply intragenic compensation.

3) Overall the methodology is clear, but I think that it would be good to have a formal methods section that describes the approaches more generally. In particular, it is not clear to me why the specific 6 sites shown in Figure 2C are shown (or if these are the differences between consensus and mouse, why the mouse and not the horse?). In general, would it be possible to devise a scheme similar to that in Figure 2C that takes into account more species, and a greater number of sites and states in those sites? I realize that the number of possible combinations increases exponentially with the number of sites, but perhaps the authors could focus on actually defining the path that evolution may have traversed in these species: removing combinations that probably have not yet been observed in evolution may help simplify the schematics. I believe that these fitness ridges are the most exciting part of this research and expanding on this would give us wonderful insights into evolution.

*Authors' response: Although we would be glad to answer positively to such an enthusiastic request, we failed to devise a proper way to draw a diagram that would take into account all the listed possibilities (more species, more sites, several states for one site). Nevertheless, we expanded this part to include methodological explanations on the general approach and to display additional examples, both simple (Figure *2A*) and more sophisticated (heptagonal diagram, Figure *4*).*

4) The question at the very end of the discussion I think is too simple. Surely, homology (orthology) is defined by common ancestry and not by the practical limitations of being able to identify it.

*Authors' response: We agree with the referee. However, our point here was not that we were not able to recognize orthology, which could be properly done. Instead, we wanted to highlight the apparent paradox posed by a subset of the signal peptides analyzed. Indeed, some of them are completely divergent in spite of being encoded by orthologous DNA fragments, yet they are strong SPs. In our opinion, they are clear examples of ****functional****convergence.*

## Reviewer's report 2

Eugene V. Koonin, National Center for Biotechnology Information, NIH, USA.

In this very interesting brief paper, Veitia and Caburet examine the conservation and divergence of the signal peptides in three sets of growth factors of the BMP/GDF family. This analysis reveals an anomalous pattern of evolution whereby the signal peptide sequences are highly conserved within mammals but are extremely divergent in non-mammal vertebrates, despite conservation of the salient properties of signal peptide. They then hypothesize that this extreme divergence involves compensatory mutations and apply a straightforward but clever approach to demonstrate the existence of such compensation by measuring the change in the quality of signal peptide prediction in different mutants. The effect of compensation indeed comes out loud and clear. This is an interesting observation of a rather general appeal (despite the fact that the analyzed data set is quite small) because rarely can compensatory amino acid replacement be demonstrated in such an explicit manner.

Although the work is interesting and certainly worth publishing, I think there are areas where considerable improvement is possible, and then, I am also confused about one of the conclusions. First potential improvements, then the confusion.

1) The signal peptides are short, so the number of mutations involved is rather small. Thus, at least in some cases, a complete enumeration of all sequences of replacements should be possible, and then, the neutral network and the attainable and prohibited paths in them can be presented explicitly. I think this would greatly increase the value of the work

*Authors' response: Indeed, this is what we now propose in our figures *2, 3* and *4*, where we provide two examples of exhaustive enumeration of the possible combinations of mutations. Figure *4* shows an almost complete neutral network, whereas the diagram in Figure *3C* displays neutral, deleterious and even slightly 'hypermorphic' mutations. Possible paths for evolution of this sequence can be deduced, starting from any single mutation and following the various lines inward, avoiding deleterious combinations. The central combination contains all mutations (i.e. it is the present state of the sequence in relevant species). We think that 7 mutations and their various combinations is the maximum that can be displayed clearly in a polygonal diagram.*

2) I realize that this is a brief Discovery Note, and yet, I think it would be most helpful to include some background, that is, what is the characteristic level of conservation divergence of signal peptides within the same phylogenetic range. That would allow the reader to better assess the novelty of the results presented in the paper.

Authors' response: We have included a table (1) displaying the % of sequence idendity for the total protein and the relevant signal peptide for the BMP/GDF family, in human/mouse and human/chicken alignments. There are several possibilities, ranging from strong conservation in the three species including the signal peptide (BMP4), passing through cases of overall conservation excluding the signal peptide (GDF8), to cases of rather low conservation in the three species (BMP15).

3) My confusion: I do not understand why the authors talk about convergence in this case, and even use exclamation points to emphasize this conclusion. As far as I know, convergence implies that similar sequences (the same amino acid residues) evolve independently in different lineages, from dissimilar ancestors. The sequences in question may well be orthologs but the essence of convergence is that they start from distant points and get closer to each other in the course of evolution (cf. the famous case of monkey lysozymes studies by A. C. Wilson and coworkers). Is this what was observed here? I did not get this impression but, if this is the case, it should be made much more transparent.

*Authors' response: The referee is right. Indeed, we should have insisted more on the fact that we were dealing with ****functional ****convergence for highly divergent sequences stemming from a common ancestral one.*
